# Caregiver support and burden drive intention to engage in a peer-to-peer exchange of services among caregivers of dementia patients

**DOI:** 10.3389/fpsyt.2023.1208594

**Published:** 2023-07-06

**Authors:** O. Zeynep Aksin, Basar Bilgic, Perihan Guner, Evrim D. Gunes, Kemal Kuscu, E. Lerzan Ormeci, Serpil Sayin, Hale Yapici Eser

**Affiliations:** ^1^College of Administrative Sciences and Economics, Koc University, Istanbul, Türkiye; ^2^Department of Neurology, Istanbul Faculty of Medicine, Istanbul University, Istanbul, Türkiye; ^3^Department of Nursing, Faculty of Health Sciences, Istanbul Bilgi University, Istanbul, Türkiye; ^4^Department of Psychiatry, School of Medicine, Koc University, Istanbul, Türkiye; ^5^College of Engineering, Koc University, Istanbul, Türkiye; ^6^Research Center for Translational Medicine, Koc University, Istanbul, Türkiye

**Keywords:** dementia caregiver support, dementia caregiver respite, decentralized allocation of caregiving time, peer-to-peer exchange of knowhow and services for caregivers, community support services, social stress, quality of life, service user engagement

## Abstract

**Introduction:**

The number of people diagnosed with dementia is increasing, creating significant economic burden globally. With the progression of the disease, patients need a caregiver whose wellbeing is important for continuous care. Providing respite as a service, through sharing the responsibility of caregiving or support for the caregiver, is a costly initiative. A peer-to-peer online support platform for dementia caregivers, motivated by the sharing economy, putting exchange of knowhow, resources, and services at its center, has the potential to balance cost concerns with a search for respite. The aim of this research is to assess caregivers’ intention to engage in peer-to-peer exchange.

**Methods:**

A survey including sociodemographic, technology use, and caregiving variables, structured questionnaires (Zarit caregiver burden, WHO brief quality of life scale, ADCS-ADL and chronic stress scale) were administered, January 2018–May 2019, in the dementia outpatient clinic of a university hospital, to a convenience sample of *n* = 203 individuals identifying themselves as primary caregivers. A path analysis exploring the drivers of an intention to engage in peer-to-peer service exchange was conducted.

**Results:**

In the path model, caregivers experiencing higher caregiver burden showed higher intention to engage (0.079, *p* < 0.001). Disease stage had no effect while patient activities of daily living, chronic social role related stressors of the caregiver and general quality of life were significant for the effect on the caregiver burden. Existing household support decreased the caregiver burden, affecting the intention to engage. Caregivers who can share more know-how demonstrate a higher intention to engage (0.579, *p* = 0.021). Caregiver technology affinity (0.458, *p* = 0.004) and ability and openness to seek professional help for psychological diagnoses (1.595, *p* = 0.012) also increased intention to engage.

**Conclusion:**

The model shows caregiver burden to be a major driver, along with caregiver characteristics that reflect their technology affinity and openness to the idea of general reciprocity. Existing support for obtaining knowhow and exchanging empathy have a direct effect on the intention to engage. Given the scarcity of caregiver support in the formal care channels, the identified potential of enlarging informal support via a peer-to-peer exchange mechanism holds promise.

## 1. Introduction

The number of people living with dementia is approximately 55 million, and it is estimated that the number will rise to 78 million by 2030, and to 139 million to by 2050 (1). The global societal costs of dementia are estimated to be US $1.3 trillion in 2019, which arise from the direct costs of social care services, and the informal unpaid inputs of family carers. Dementia is a chronic condition for which there is currently no cure, even though there are very recent treatments to potentially delay cognitive decline in Alzheimer’s disease, a most common form of dementia (2, 3). As the disease progresses, people with dementia will need increasing support. Until a cure is found, the global need for care will continue to rise. The purpose of this research is to explore the potential of expanding caregiving supply via an exchange between caregiving peers. The idea of such peer-to-peer exchange has its roots in the sharing economy. The current study explores whether there is a segment of dementia caregivers who are willing to engage in a possibly reciprocated exchange of caregiving services with their peers.

The growth in the population with dementia outpaces the capacity of formal care services, necessitating a larger portion of unpaid care provided by family members and wider family networks. Known as informal care, the costs associated with primary caregivers in this category constitute the indirect costs of dementia. Informal care includes activities such as assisting the patient with basic and instrumental activities of daily living (ADLs) and patient supervision, while costing informal care is generally based on an opportunity cost approach (4). In a study based in the UK, the indirect costs associated with dementia are almost equal in magnitude to direct costs which consists of medical costs and social care costs (5). The costs of informal care are more dominant in low and middle-income countries (6).

Dementia caregivers constitute a vulnerable segment in the society due to the psychosocial impact of caring for dementia patients. In addition to the physical load in terms of hours of care provided, dementia caregivers experience the cognitive decline of loved ones, and this is a source of psychological distress for them (7). Institutionalization of dementia patients does not necessarily improve the wellbeing of caregivers and they may need to be supported during the transition experience (8). Supporting the caregiver throughout the process of informal care is essential to ensure their wellbeing (9). This has led to extensive research on interventions for dementia caregivers’ wellbeing, ranging from cognitive-behavioral therapy, counseling, social support to respite, where respite is defined as the enabling of short breaks away from caregiving responsibilities. Interventions such as psychoeducation and cognitive-behavioral therapy mildly decrease caregiver burden, depression and anxiety, while moderately increasing the ability and knowledge of the caregivers (10). Interventions that combine more than one approach are found to be more effective.

Respite services take the form of some means to share responsibility of caregiving or some form of support for the caregiver (day care center, help for home care, etc.). Providing respite as a service is a costly initiative. A qualitative study focusing on respite care offered in developed countries indicates the following barriers to utilization of such services: the inability to find information about relevant services, the poor quality or mistrust of the services, the inflexibility of services, caregivers’ beliefs about their obligations to the caregiving role and resistance by the care recipient (11). In a review (12), caregivers are found to associate seeking respite with the feeling of evasion of responsibility and to seek internal respite services provided by friends and family more easily than they seek external respite services. A peer-to-peer exchange of caregiving services can be positioned as a form of internal respite and may provide an alternative that enables respite while overcoming some of its typical barriers. Nevertheless, quality and trust issues may remain.

State initiatives for caregiver support, sponsorship or donations to non-profit societies for a large-scale intervention, are lacking in many countries. Focusing on the funding aspect of support for family caregivers, Teahan et al. (13) show that the public in Ireland is willing to pay additional taxation to support family caregivers. Such support may not be feasible in less developed countries. Many families are unable to afford services offered by for-profit alternatives in the marketplace, implying a need for a system that can provide support to many caregivers without charging them for a fee. The sheer magnitude of the problem far exceeds formal care channels. A solution to this problem can only be meaningful if it can be scaled up easily to the large and growing population of dementia patients and their caregivers.

Developments in Information Communication Technologies, along with changes in consumer attitudes, have recently enabled the formation of new businesses in what is labeled as the sharing economy. Their business models involve systems of organized sharing, bartering, lending, trading, renting, gifting and swapping (14). Uber and Airbnb are among the most well-known representatives of the sharing economy. We believe that a peer-to-peer support platform as described below has the potential to balance cost concerns with a search for caregiver respite. The current study aims to explore the willingness for peer-to-peer exchange in the dementia caregiving domain, as a first step for the future design of a platform.

To the best of our knowledge, a peer-to-peer service system that addresses caregivers’ respite needs does not exist. There are online resources to support caregivers’ need to seek information and connect with peers, which are found to be effective (15). The platform we envision differs from those resources by going beyond online support to the consideration of services potentially involving the care recipient (the dementia patient), in a peer-to-peer setting, not involving any monetary exchange. Some existing platforms (such as lotsahelpinghands) aim to address the communication and coordination needs of a patient’s care team; however, they do not facilitate exchange and collaboration between different care teams. The goal of a respite platform would be to bring together communities of dementia caregivers, families, and volunteers for an exchange of support. This platform would allow matching the demand for social support, support for various errands such as shopping, cooking, help with caregiving activities, and short-duration day care with services that are provided again by members of the community.

Among existing peer-to-peer systems, time banking and hospitality exchange platforms provide benchmarks along certain features. Time banking systems involve an exchange of services among participants in a social network, where all services are evaluated in terms of time as a common currency. Commitment in time banking systems is shown to come in different forms. Users that are active in both give and take are critical, and it is found that the success of a time bank depends on this general reciprocity (16). Hospitality exchange platforms require commitment to general reciprocity as well as offline interactions among peers, making trust and intimacy a central component (17, 18). The conceptualized peer-to-peer platform aims to create an exchange of needs and resources between households, which can be seen as an exchange of caregiving time. Households can be loosely defined as members of family and friends of a caregiver who do or may potentially contribute to the caregiving. The exchange may necessitate offline interactions of caregivers and care recipients in their homes, thus requiring high levels of trust and security. The endeavor of a new digital platform design falls in the domain of design science research. According to the design science research methodology put forth by (19) our research lies at the initial phases, covering the problem identification and motivation step and a first attempt to define the objectives of the solution. We seek to identify the existence and features of caregiver households who would be willing to actively supply and demand support for caregiving.

The envisaged platform has the potential to create value by enabling pooling of resources of different households (20, 21). It also provides an opportunity to create complementarities between individual caregiver capabilities, resources and needs that may arise mainly due to dynamics of disease progression. The platform can allow for exchange of caregiving related services, exchange of expertise and knowhow, as well as exchange of appreciation and social support leading to community building. A sense of community can further enhance trust and intimacy in the social network of caregiving households, in turn creating the required ecology for exchange (22–24). According to this system description, basic drivers of value generation are determined by the needs of caregivers, the needs of the dementia patient, their household context and existing support, as well as some of their personality features. Value can only be created if individual caregiving households are willing to engage in exchange via such a platform. System level feasibility and sustainability hinges on individual level willingness or openness to become active members and users of such a platform.

**Figure 1:**
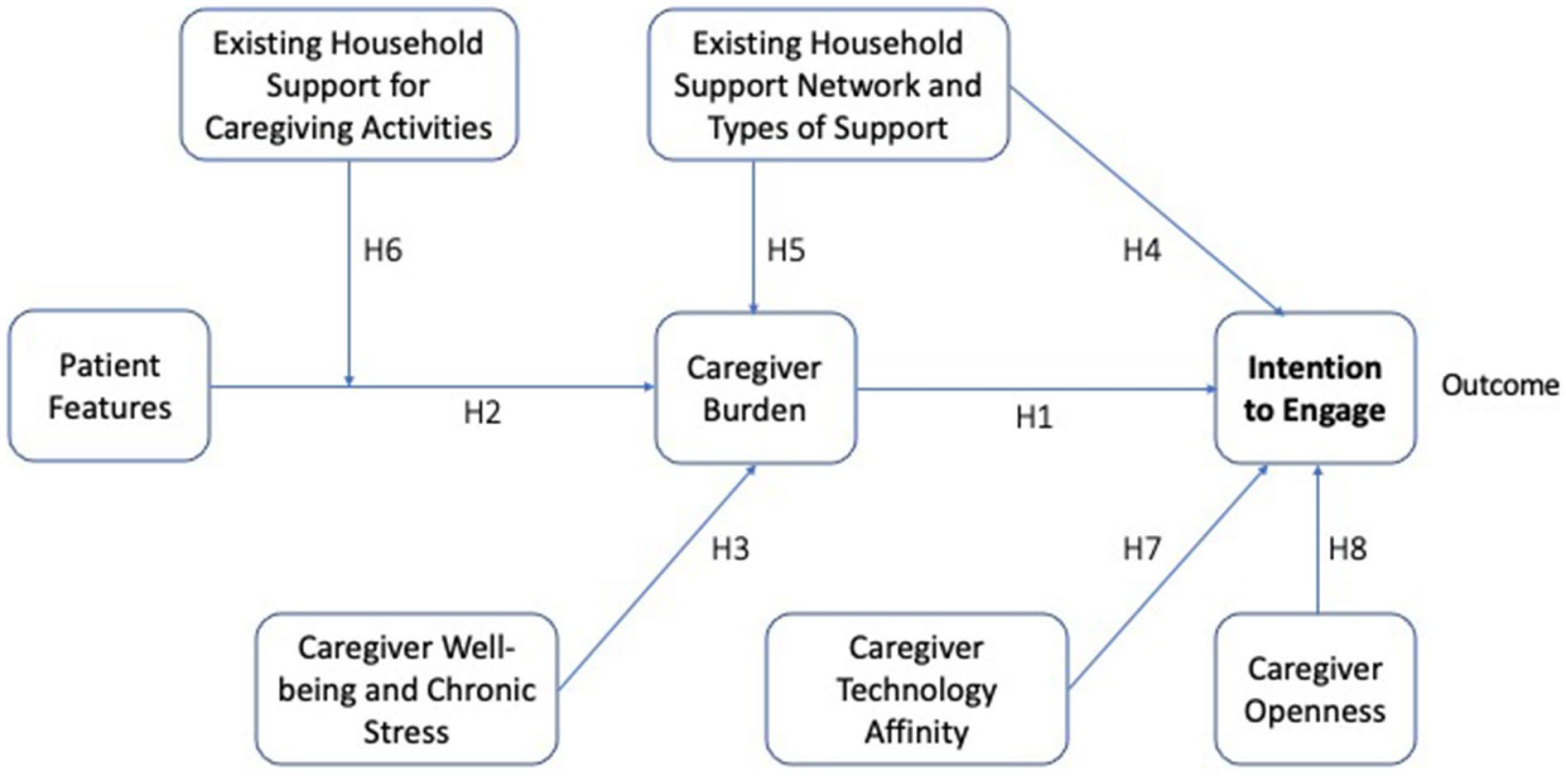
Conceptual model. Conceptual framework of direct and indirect associations between patient features (needs), caregiver needs (well-being and chronic stress), existing household support, caregiver characteristics (technological affinity and openness) and the intention to engage in a peer-to-peer caregiver support platform. Caregiver burden is a mediator of the association between patient features and household context and intention to engage, whereas the household context moderates the association between patient features and caregiver burden. Hypothesis numbers are given on the associated links.

The aim of the current research is to study the variables that will influence the intention to engage in a reciprocated exchange of caregiving related knowhow, support, and resources among dementia caregivers. For this aim, we explored caregivers’ existing support networks, their needs and intentions for engagement in exchange. We analyze the *intention to engage*, without providing a context in terms of any digital platform features. The purpose is to assess general tendencies for peer-to-peer exchange. We seek answers to the questions: are there caregivers who express an intended engagement of their household as demand generators or service providers within such an exchange? What are the characteristics of such households? What is the role of heterogeneity along caregiver demographics and patient characteristics? We explore these questions with a path analysis testing the relations shown in Figure 1. (The variables and hypotheses shown on the figure are developed and described in the Materials and Methods Section below.) This constitutes a first step in exploring the feasibility of the proposed peer-to-peer exchange from the perspective of individual caregivers and their households.

## 2. Materials and methods

### 2.1. The survey

We pursued a survey methodology with a cross-sectional design. An *a priori* power analysis making use of G*power version 3.1.9.2, using medium effect size, power of 0.80, alpha of 0.05, the required sample size was found to be 181. The survey was administered between the dates January 2018 to May 2019, in the dementia outpatient clinic of a university hospital to a convenience sample of individuals who identified themselves as primary caregivers of patients. Inclusion criteria were being a primary caregiver, being 18 years or older, and being proficient in Turkish. Exclusion criteria was having a serious psychiatric or cognitive function disorder (unable to read and comprehend and respond). Participants signed an informed consent form before taking the survey. *n* = 203 caregivers participated in the survey. Nine participants were excluded due to excessive missing information (more than 90%). Paid caregivers (10 participants) were excluded. The analysis reported below is based on *n* = 184 caregivers. At the time of the study, the clinic served around 800 active patients. It is one of the major referral centers for dementia patients in the city, thus providing a more heterogenous cross-section compared to an average clinic of the same size. The sample represents nearly a quarter of the population of this clinic.

The survey was administered in person in the waiting area of a clinic, after completion of the medical appointment. All surveys were conducted by two surveyors with degrees in clinical psychology, who were trained to perform the surveys by posing the questions as written (to ensure inter-rater reliability), adopting a neutral tone without affect. To avoid respondent fatigue and enable completion in a reasonable amount of time, the survey time was limited to around forty-five minutes. Existing scales were used for the most important variables measuring caregiver and patient features, while keeping the social context, technology, demand and supply for support related parts in independent questions. The survey did not refer to a platform or any other mechanism, and all questions about existing support, demand for help from others, supply of help to others and expectations about exchanging help with other households were posed without a context.

The survey is coping and load focused, due to its attempt to take a first step in understanding demand and supply for reciprocated support. The first part of the survey contains 34 independent questions including demographics (age, gender, education, marital status, employment status, income level), technology use (use of smartphone, internet access, use of social networking apps, use of online shopping, use of online services), caregiving context (relationship to patient, number of adults in household, relationship of adults in the household to caregiver, caregiving duration in years, number of hours of caregiving per day). (An English translation of these questions are provided as Supplementary material.) The following variables are all measured on a Likert scale of 5 (never-always): the nature and amount of support the caregiver has in terms of types of support (ability to entrust the patient to someone for a limited duration, help with caregiving tasks, help with household chores, obtaining knowhow regarding caregiving, opportunity to share and exchange caregiving related problems and feelings), channels through which these are obtained (from family members, from neighbors and friends, from relatives, from paid helpers, from official sources), intention to seek help (broken up by type of support and channel of support in terms of support from professionals versus peers), intention to provide help to others (personal intention, intention on behalf of family member). Expectations on the nature of exchange for such help (expect nothing in return, expect equivalent service in return, expect pay for service if any), caregiver health (physical and psychological diagnoses, their diagnosis timing and nature).

The second part of the survey consists of structured questionnaires that measure caregiver perceived burden [ZBI Zarit caregiver burden (25, 26)], quality of life [WHOQL-brief quality of life scale (27, 28)], patient daily activities ADCS-ADL (29), social roles related stress [chronic stress (30, 31)]. Patient files were consulted to obtain clinical dementia rating (CDR) scores of patients. All the patients or their legal representatives provided consent for the use of clinical data in scientific studies upon initial visit to the University Clinic.

### 2.2. The path model and the hypotheses tested

Path analysis is an extension of multiple regression. It is useful when there are more than one dependent variables, and when some variables may act as both independent and dependent variables with respect to other variables (32). For our model, since the variable caregiver burden is a dependent variable which also acts as an independent variable for intention to engage, path analysis is appropriate. Our study adopts a cross-sectional design, so causal relationships between variables need to be interpreted with caution. These should be further explored in future work with longitudinal data. All analysis is performed making use of R version 4.2.1. We employ the Lavaan package (version 06-12) for the path analysis. To operationalize the main outcome variable, intention to engage, we focus on caregivers who would show affinity to peer-to-peer exchange by measuring their intended demand for support from peers, intended supply for support to peers by them and their household members, and willingness to reciprocate, via survey questions.

The ability to pool knowledge and resources via household-to-household exchange hinges on caregiver needs, patient needs, household context and needs, and caregiver heterogeneity. Sustained exchange will require a sufficiently high volume of demand and supply for support opportunities to ensure a possibility for reciprocity.

#### 2.2.1. The effect of caregiver burden

The effect of dementia on caregivers is captured by constructs of caregiver burden. A vast literature studies caregiver and patient determinants that drive subjective caregiver burden (33, 34). Among patient features, lack of self-care and need for support are features that are expected to directly affect caregiving needs. Support and respite care are seen as instruments to address caregiver burden (35, 36). A peer-to-peer exchange for caregiving support and respite care can act as such an instrument and be perceived as useful by caregivers experiencing caregiving burden. Viewing the platform as technology, the intention to engage in a peer-to-peer exchange can be seen from the perspective of technology acceptance. The technology acceptance model (TAM) (37) has been proposed to study the intention to use a technology, relating adoption of the technology to its perceived usefulness and perceived ease of use. TAM has been expanded to capture a health behavior perspective when studying acceptance of mobile health services (38), and personal characteristics of users and their perceived risk when applied in a sharing economy setting (39). Unlike systems studied in the technology acceptance literature, the platform does not exist and is only articulated at a conceptual level. For this reason, we do not test the TAM, but in line with its perceived usefulness driver, hypothesize that the need for support will drive the intention to engage in the conceptualized exchange. We expect the demand for services via the proposed online platform, and thus the intention to engage, to be affected by caregiver burden. The platform we study has a healthcare domain focus. Furthermore, the user in our study is not the care recipient but a caregiver associated with a care recipient and their household. These features suggest the need to capture the exchange nature of a sharing platform as well as antecedents that depend on both sides of the care recipient-caregiver dyad, which is not part of the TAM setting. We expect caregiver perceived burden to mediate care recipient and caregiver determinants on the intention to engage. The three hypotheses tested on this background were as follows:

**H1:** *Caregiver burden characterizes the need for support from a peer-to-peer support platform and increases intention to engage.*

**H2 (Mediation):** *The effect of care recipient features on intention to engage is mediated by caregiver perceived burden.*

**H3 (Mediation):** *The effect of caregivers’ general wellbeing and chronic stress on intention to engage is mediated by caregiver perceived burden.*

#### 2.2.2. The effect of existing caregiving support

The existing support that caregivers receive from their households establishes part of the caregiving context and is an important driver of subjective caregiver burden (40, 41). Support can take different forms [psychoeducational, respite, general support (10)]; (1) information support or the ability to obtain recommendations and knowhow related to caregiving, (2) ability to obtain help with caregiving activities, (3) ability to entrust the patient to others for short durations, (4) help with other household chores, and (5) the ability to share or talk about problems with others. The peer-to-peer platform is envisioned as having a design where services are exchanged along these five key dimensions.

An important aspect of our analysis is the separate measurement of these different types of support, and hypotheses about the direct as well as indirect role these play on the intention to engage. Different forms of caregiver support can affect caregiver perceived burden and thereby have an indirect effect on intention to engage. The direct effect may differ by type of support. For example, having support related to caregiving activities or household chores, and being able to entrust the patient to others, will potentially reduce the need for help beyond a caregiver’s household, thereby reducing intention to engage. In contrast, support that enables exchange of knowledge or empathy would enhance a positive attitude towards general reciprocity. The exchange of knowledge or empathy can be seen as a form of internal support (42), enhancing a sense of mastery and self-efficacy. Increased self-efficacy in turn is aligned with an increased intention to engage (43, 44). We expect existing support to have a possibly dual effect, one directly on the intention to engage and one via caregiver perceived burden, while the sign of the overall effect may vary by the type of support. The three hypotheses 4 to 6 tested on this background were as follows:

**H4:** *Existing household support of different types increase/decrease intention to engage.*

**H5 (Mediation):** *Caregiver perceived burden partially mediates the effects of existing household support on intention to engage.*

In line with earlier literature (45) we hypothesize that caregiver support for caregiving tasks moderates the impact of the care recipient determinants on caregiver burden. The care recipient’s capabilities in terms of autonomy in daily living activities drive caregiver load. Support that helps caregivers in assisting care recipients with such daily living activities can moderate the perceived burden by caregivers.

**H6 (Moderation):** *Existing household support on caregiving activities moderates the impact of care recipient characteristics on caregiver burden.*

#### 2.2.3. The effect of caregiver personal characteristics

Caregivers who demonstrate more self-efficacy and who are more willing to invite alternative sources for support or provide voluntary support to others, would seek help from others outside their current networks. The envisaged online platform also has a technology aspect to it, requiring openness to the use of technology by caregivers. The literature on technology acceptance highlights personal innovativeness in information technology as an important individual difference variable, defined as the general willingness to try out new information technology (46). In addition to personal characteristics, in a peer-to-peer setting, perceived risk is shown to affect intention of use (39). Some of the perceived risk can be related to trust among peers who may not know each other and who transact in a digital platform setting. Mohlmann (47) states that collaborative consumption on peer-to-peer platforms goes beyond standard technology use, due to the exchange of services among peers nature, and digital trust (confidence in a secure digital world) is said to play a pivotal role. It is argued that digital trust is harder to achieve on peer-to-peer collaborative consumption platforms, compared to first-generation platforms like eBay or e-commerce platforms. A hierarchical trust construct, where trust in the platform serves as a mediator for trust in peers in the context of peer-to-peer sharing platforms, is theorized and empirically supported in that paper. We do not measure self-efficacy, personal innovativeness, or digital trust due to the lack of an existing system. However, we use proxy measures that are intended to differentiate caregivers’ personal characteristics, revealed by their choices in their daily use of technology instead. These proxies can be seen as a reflection of the self-disclosure characteristics of caregivers. Use of online shopping, use of online services, or use of social media platforms are such measures, indicating operational and pragmatic caregivers, showing self-efficacy (48), personal innovativeness and demonstrating digital literacy and trust in various contexts. We refer to these proxy measures as *caregiver technology affinity*. Being open to the idea of getting help indicates an ability to understand one’s own problems as a caregiver and to seek solutions for these. Once again, we do not have a direct measure of openness but are looking for a proxy measure via a revealed choice that signals a caregiver who has acknowledged being overburdened and has asked for help. Seeking professional help for psychological diagnoses is a proxy measure, that can serve as an indication of a caregiver who can mentalize the illness and who demonstrates self-disclosure and a willingness to seek help in a situation of vulnerability where needed.

**H7:** Caregiver technology affinity increases intention to engage.

**H8:** Caregiver ability or openness to seek professional help for psychological diagnoses increases intention to engage.

These hypotheses lead to the path model depicted in Figure 1 in the previous section.

Our aim is to understand the drivers of the intention to engage in a peer-to-peer caregiver support and respite platform. The measure for intention to engage needs to capture the notion of general reciprocity. This means that demand, supply, and their exchange should be included. We operationalize intention to engage by making use of demand for support from peers, intention to provide similar services to peers (by caregiver or by caregiver’s household members), and via the exchange mechanism that captures general reciprocity, i.e., in return for similar services. The intention to engage variable, ENGAGE is thus defined as the sum of three terms: (1) the average of demand for support from peers over the five categories of support (entrusting patient, help with caregiving tasks, help with household chores, sharing knowhow, sharing experiences and empathy), (2) the average over the five categories of support of the maximum of own or household member intention to supply, and (3) the intention to exchange services for similar services. The variable takes values between 3 and 15 and has acceptable reliability (mean 9.37, SD 3.2, *N* = 184, *α* = 0.87).

Caregiver burden (Zarit burden index) is the second endogenous variable, which mediates the effect of patient features, caregiver wellbeing, and the existing support network. The variable has acceptable reliability (mean 36.84, SD 16.77, *N* = 184, *α* = 0.89).

Exogenous variables fall under three main categories, pertaining to the caregiver, the caregiving context or household, and to the patient. Some of the variables are directly measured via survey questions or existing scales: caregiver and household demographics (age, gender, education, marital status, employment status, income level), caregiver wellbeing (WHQOL-BREF quality of life scale), caregiver stress (chronic stress scale), patient daily living capabilities (ADCS-ADL), patient disease stage (CDR score).

Existing household support is operationalized in four variables, one for each category of caregiving related support: (1) ENTRUST: entrusting the care recipient, (2) CARE: help with caregiving tasks, (3) KNOWHOW: sharing know-how, and (4) EMPATHY: sharing experiences and empathy. The support score for each category is calculated as an average of the scores (Likert scale of five) for support received from the three channels (family members, neighbors and friends, relatives), in line with the envisaged peer-to-peer nature of the platform. The two support categories, ENTRUST and CARE, showed high correlation (0.79) in the sample, hence only one of these is included in the final model. Entrusting the care recipient implies the absence of the caregiver and describes a type of support that requires high levels of trust and familiarity between the caregiver and the person providing the support. On a peer-to-peer platform, this may be difficult to envision or ensure early on, so we keep the support for caregiving tasks in the model. Directionally and in terms of statistical significance, results are shown to be robust to the choice of CARE versus ENTRUST in the model.

We consider social network application, online shopping, and online service use, as possible variables to operationalize caregiver technology affinity. Online shopping and online services can be considered as demonstrating higher digital literacy, so we prefer these variables. In the sample the two variables are highly correlated (0.785). Some of the caregivers in this clinic use an online appointment system and report this as use of online services, so they are less differentiated along this dimension. We consider online shopping as a proxy for caregiver technology affinity. Directionally and in terms of statistical significance, the results are robust to the choice between online services and online shopping. Binary responses to a question on seeking psychological help from professionals act as a proxy for caregiver openness.

## 3. Results

Table 1 presents descriptive statistics for the sociodemographic variables as well as other caregiver/household related variables used in the analysis. A reported percentage indicates that the associated variable is a dummy variable. Detailed descriptions of these variables, if applicable, are given in Table 2. In analysis not reported for brevity, Variance Inflation Factors for the variables used in the final models are shown to be less than 2 with only one taking a value higher than that (but still less than 10), alleviating concerns for multicollinearity.

**Table 1:** Descriptive statistics for sociodemographic and caregiver/household related variables.

Feature	Descriptive statistics
Age	Mean 52.61, Std. dev. 12.05
Gender	29% (54) male, 71% (130) female
University degree	65% (119)
Married	71% (130)
Employed	28% (52)
High income level	10% (18)
Online shopping	Mean 2.08, std. dev. 1.57
Seek psychological help	12% (21)
Full time caregiver	67% (124)

**Table 2:** Detailed descriptive statistics for sociodemographic variables.

Education	*n*
1: Illiterate	3
2: Literate	4
3: Primary school	47
4: Secondary school	11
5: High school	53
6: College	53
7: Masters	11
8: PhD	2

Marital status	*n*
1: Single	35
2: Married	130
3: Widow/divorced	19

Income	*n*
1: Very low	5
2: Low	22
3: Middle	139
4: High	18

Occupation	*n*
1: Full time job	44
2: Part time job	8
3: Housewife	52
4: Student	0
5: Retired	54
6: Quit work for caregiving	9
7: Other	14

Caregiving since	*n*
1: Less than 1 year	16
2: 1–2 years.	42
3: 3–5 years	68
4: 6–10 years	37
5: 11 years and more	21

Caregiving time per day	*n*
1: 0–1 h	11
2: 2–3 h	14
3: 4–5 h	15
5: 6–8 h	20
6: 9–24 h	124

Number of adults in household	*n*
1	44
2	64
3	32
4	28
5	16
Patient is spouse of caregiver	61 (33%)
Patient is first degree relative of caregiver	162 (88%)
Employs fulltime paid help	17 (9%)
Smartphone ownership	157 (85%)
Internet access	142 (77%)

Online services use	*n*
1: Never	99
2: Rarely	12
3: Sometimes	9
4: Frequently	18
5: Always	42

Table 3 tabulates means and standard deviations for existing support variables (ranging from 1–5 where 1 indicates “never” and 5 indicates “always”) and the intention to engage variable (ranging from 3–15, calculated as the sum of three variables ranging between 1–5, indicating a range from “never” to “always”). The mean values show that in the sample existing support is low, taking the highest value along the sharing empathy and experiences dimension. The mean of the intention to engage variable is in the higher half of the range. Table 4 tabulates variable minimum-maximum values, medians, means, standard deviations, and Cronbach’s Alpha for the measured scales.

**Table 3:** Descriptive statistics for existing support variables and the intention to engage variable.

Feature	Mean	Standard deviation
Support: entrusting the patient (ENTRUST)	2.37	0.94
Support: help with care (CARE)	2.30	1.02
Support: sharing know-how (KNOWHOW)	1.47	0.92
Support: empathy (EMPATHY)	3.00	1.30
Intention to engage (ENGAGE)	9.37	3.20

**Table 4:** Descriptive statistics and Cronbach’s *α* for measured scales.

Scale	(Min, max)	Median	Mean	Std. dev.	*α*
ZBI	(0, 84)	36	36.84	16.77	0.89
Chronic stress	(3, 60)	25	25.75	12.93	0.86
ADL score	(0, 78)	22	27.20	22.55	
CDR score	(0.5, 3)	2	1.67	0.96	
QOL general	(2, 10)	6	6.25	1.58	0.8

**Figure 2:**
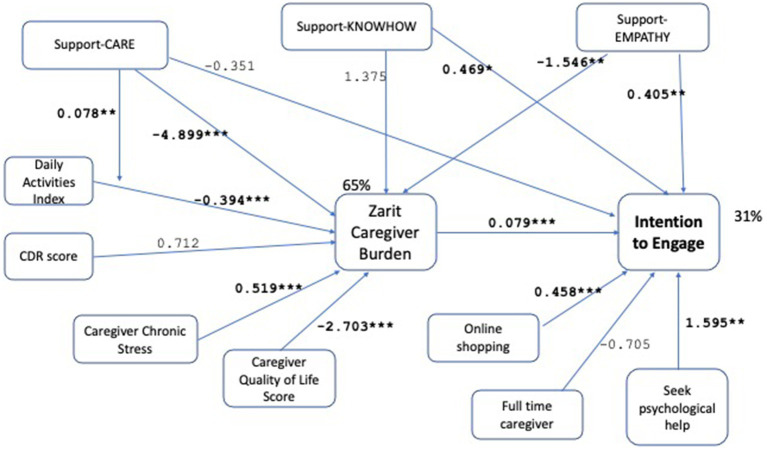
Path model analysis results with unstandardized path coefficients. The model demonstrates good fit with *χ*^2^ (*p*-value = 0.941 > 0.05), RMSEA (RMSEA ≤0.05 *p*-value = 0.983), CFI (1.0 > 0.9), SRMR (0.006 < 0.08) measures. The significant associations are shown bold with the coefficients shown on the links, and the significance levels indicated as (^*^p < 0.1, ^**^p < 0.05, ^***^p < 0.001). The *R*-square values are 65% for caregiver burden, 31% for the intention to engage. The conceptual model shown in Figure 1 is operationalized with the variables in the model above. Thus, for each concept there is more than one measure used; for example, existing household support is measured by support-CARE, support-KNOWHOW, support-EMPATHY. The model supports all tested hypotheses. While some of the measures for each concept remain insignificant, there is at least one measure for each concept that shows a significant association supporting the stated hypothesis.

**Figure 3:**
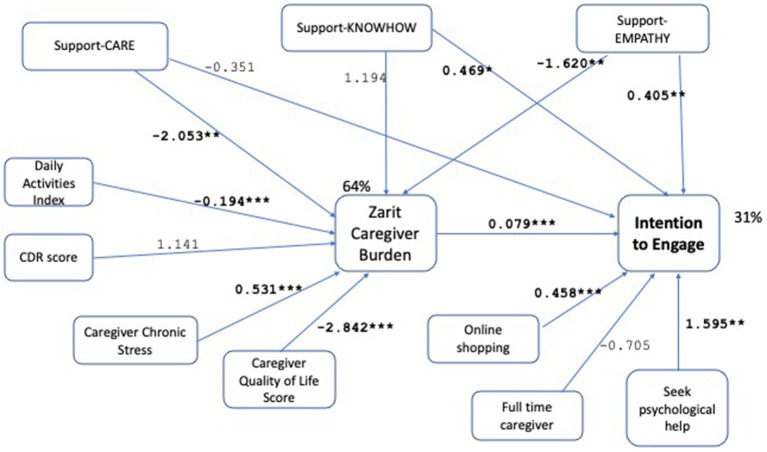
Path model without moderation effect, results with unstandardized path coefficients. Demographic controls are omitted from the figure for clarity of presentation. ^*^p < 0.1, ^**^p < 0.05, ^***^p < 0.001. This model is a robustness check for the model given in Figure 2, where the only modification is the removal of the moderation of daily activities by support-CARE. The model again demonstrates good fit with *χ*^2^ (*p*-value = 0.925 > 0.05), RMSEA (RMSEA ≤0.05 *p*-value = 0.983), CFI (1.0 > 0.9), SRMR (0.006 < 0.08) measures. *R*-square for caregiver burden and intention to engage are found as 64% and 31%, respectively. All hypotheses are supported by significant associations with at least one measure, supporting robustness of the results of the original model (given in Figure 2).

**Figure 4:**
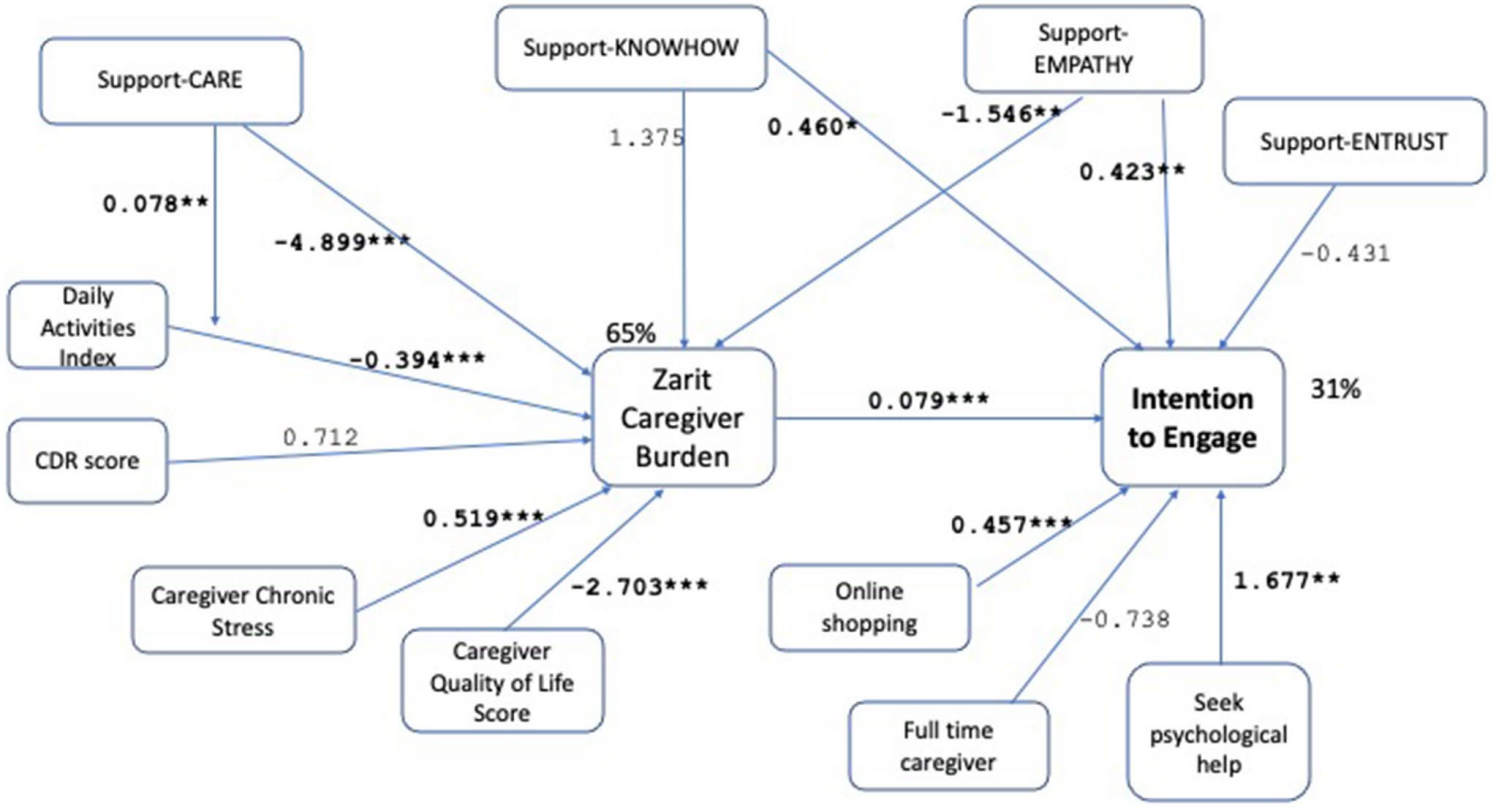
Path model with support-entrust replacing support-care in the intention to engage regression. Results with unstandardized path coefficients. Demographic controls are omitted from the figure for clarity of presentation. ^*^p < 0.1, ^**^p < 0.05, ^***^p < 0.001. The model demonstrates good fit with *χ*^2^ (*p*-value = 0.975 > 0.05), RMSEA (RMSEA ≤0.05 *p*-value = 0.995), CFI (1.0 > 0.9), SRMR (0.007 < 0.08) measures. *R*-square for caregiver burden and intention to engage are found as 65% and 31%, respectively. All hypotheses are supported by significant associations with at least one measure, supporting robustness of the results of the original model (given in Figure 2).

**Figure 5:**
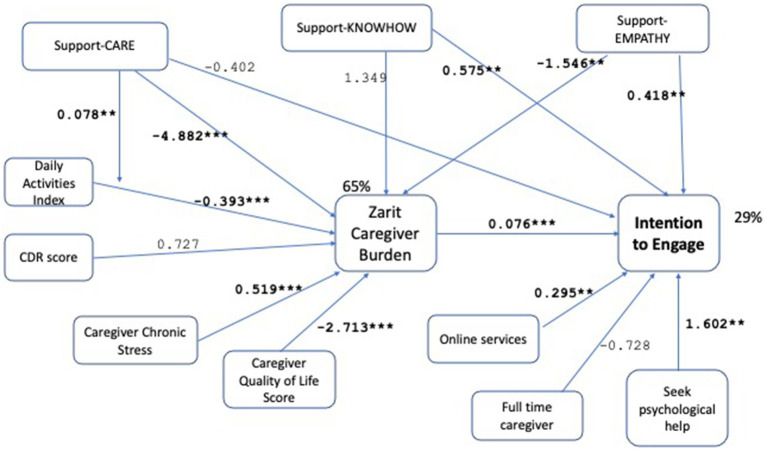
Path model with online services replacing online shopping in the intention to engage regression. Demographic controls are omitted from the figure for clarity of presentation. ^*^p < 0.1, ^**^p < 0.05, ^***^p < 0.001. The model demonstrates good fit again with *χ*^2^ (*p*-value = 0.906 > 0.05), RMSEA (RMSEA ≤0.05 *p*-value = 0.971), CFI (1.0 > 0.9), SRMR (0.007 < 0.08) measures. *R*-square for caregiver burden and intention to engage are found as 65% and 29%, respectively. All hypotheses are supported by significant associations with at least one measure, supporting robustness of the results of the original model (given in Figure 2).

We test the hypotheses by operationalizing the path model in Figure 1, leading to the final model shown in Figure 2. Demographic controls are omitted from the Figure for clarity of presentation. The model is evaluated as demonstrating good fit with *χ*^2^ (*p*-0.941 > 0.05), RMSEA (RMSEA ≤0.05 *p*-value 0.983), CFI (1.0 > 0.9), SRMR (0.006 < 0.08). For a detailed tabular view of the model please refer to Table 5. Robustness checks with alternative variable choices, without the moderation effect in H5, with support-entrust replacing support-care, with online services replacing online shopping, are shown in Figures 3–5 respectively, similarly demonstrating good fit.

**Table 5:** Path model results from Lavaan package in R.

Model	
Test statistic	2.883
Degrees of freedom	8
*p*-value (chi-square)	0.941
RMSEA	<0.001
90% CI-lower	0
90% CI-upper	0.018
SRMR	0.006

ZBI	Estimate	Std. err.	*z*-value	*p*(>|*z*|)	Std. all
*Regressions*					
Age	0.084	0.08	1.054	0.292	0.06
Male	−2.338	1.864	−1.254	0.210	−0.063
University	0.68	1.815	0.375	0.708	0.019
Married	−7.184	1.889	−3.804	0.000	−0.194
Works	3.086	1.965	1.57	0.116	0.083
High income	3.295	2.748	1.199	0.230	0.059
CARE	−4.899	1.466	−3.343	0.001	−0.298
KNOWHOW	1.375	0.904	1.52	0.128	0.077
CARE:ADL	0.078	0.031	2.501	0.012	0.337
EMPATHY	−1.546	0.688	−2.245	0.025	−0.118
ADL	−0.394	0.092	−4.286	0.000	−0.531
CDR	0.712	1.037	0.686	0.493	0.04
Chronic stress	0.519	0.078	6.67	0.000	0.397
QOL general	−2.703	0.633	−4.271	0.000	−0.249
*ENGAGE*					
ZBI	0.079	0.014	5.623	0.000	0.427
Age	0.008	0.02	0.413	0.680	0.032
Male	0.273	0.478	0.572	0.567	0.04
University	0.325	0.499	0.652	0.515	0.049
Married	0.635	0.488	1.303	0.193	0.092
Works	0.154	0.544	0.283	0.777	0.022
High income	1.307	0.712	1.836	0.066	0.125
Online shopping	0.458	0.159	2.876	0.004	0.229
Full time caregiver	−0.705	0.527	−1.338	0.181	−0.107
KNOWHOW	0.469	0.242	1.942	0.052	0.141
EMPATHY	0.405	0.178	2.277	0.023	0.166
Psychological help	1.595	0.638	2.499	0.012	0.167
CARE	−0.351	0.244	−1.435	0.151	−0.115
*R-Square*					
*Estimate*					
ZBI	0.648				
ENGAGE	0.305				

Defined parameters					
	Estimate	Std. err.	*z*-value	*p*(>|*z*|)	Std. all
Indirect KNOWHOW	0.109	0.074	1.468	0.142	0.033
Direct KNOWHOW	0.469	0.242	1.942	0.052	0.141
Total KNOWHOW	0.579	0.251	2.309	0.021	0.174
Indirect EMPATHY	−0.123	0.059	−2.085	0.037	−0.05
Direct EMPATHY	0.405	0.178	2.277	0.023	0.166
Total EMPATHY	0.282	0.184	1.531	0.126	0.116
Indirect CARE	−0.389	0.135	−2.873	0.004	−0.127
Direct CARE	−0.351	0.244	−1.435	0.151	−0.115
Total CARE	−0.74	0.262	−2.821	0.005	−0.242

We find support for Hypothesis 1 showing that caregiver burden is an important driver of *ENGAGE*, with those experiencing higher care burden showing higher intention to engage (0.079, *p* < 0.001, Figure 2). In addition to the strong statistical significance shown by the small *p*-value, the practical significance of the effect needs to be evaluated. For instance, if the care burden increases by one standard deviation, the expected change in ENGAGE is 16.77 × 0.079 = 1.3. Remembering that ENGAGE takes values between 3 and 15, this can be considered as a practically significant effect. The standardized coefficient of the variable in Table 5 (0.427) indicates a meaningful effect size. Hypothesis 2, which states a mediation by caregiver burden of patient features, is supported for activities of daily living, while the stage of the disease captured by the CDR score is not significant. Caregivers of care recipients with higher capabilities in daily living activities exhibit lower perceived caregiver burden (−0.394, *p* < 0.001, Figure 2). Both Chronic Stress (0.519, *p* < 0.001, Figure 2) and general quality of life (−2.703, *p* < 0.001, Figure 2) variables capturing the general wellbeing of caregivers are significant and provide support for Hypothesis 3 (Figure 2). In addition to high statistical significance, the standardized coefficients in Table 5 show medium to high effect sizes for these variables (−0.53 for ADL, 0.39 for chronic stress, −0.25 for QOL general). Higher chronic stress increases the perceived caregiving burden while higher general quality of life decreases it. Among the demographic control variables for caregiver burden (age, gender, education, married, working, income), only being married is significant (−7.184, *p* < 0.001). The effects of patient features (patient daily activities) and caregiver general wellbeing (QOL General, negative of chronic stress) on reducing burden, confirm results from the literature (33, 49) and act as a control here.

The variables that characterize existing support of the caregiver are shown to have different effects on the intention to engage. The total effect of *CARE*, which can be calculated as the sum of the direct and indirect effects is significant but negative (−0.740, *p* = 0.005, Figure 2). Caregivers who receive help with caregiving tasks experience lower burden as shown by the negative and significant indirect effect (−4.899 × 0.079 = −0.387, *p* = 0.004, Figure 2). The direct effect of *CARE* on the intention to engage is not significant (−0.351, *p* = 0.151, Figure 2). We observe a significant total effect with a negative coefficient (−0.740, *p* = 0.005, Figure 2) suggesting that caregivers who receive help with caregiving tasks cannot envisage further benefit from receiving additional support along this dimension. Hypothesis 4 (decrease) and Hypothesis 5 are thus supported for *CARE*. The moderating effect of this variable on daily living activities is also significant (0.078, *p* = 0.012, Figure 2), supporting Hypothesis 6. The existing support for *KNOWHOW*, is significant and positive, indicating that caregivers who can share more knowhow demonstrate a higher intention to engage (0.469 + 0.109 = 0.579, *p* = 0.021, Figure 2). This effect is driven by the direct effect of such support (0.469, *p* = 0.052). The indirect effect is not significant (1.375 × 0.079 = 0.109, *p* = 0.142, Figure 2). Hypothesis 4 (increase) is supported while Hypothesis 5 is not for *KNOWHOW*. Finally, *EMPATHY* appears insignificant for the total effect (0.405–0.123 = 0.282, *p* = 0.126, Figure 2). This is due to the significant and opposing direction of the direct (0.405, *p* = 0.023, Figure 2) and indirect (−1.546 × 0.079 = −0.123, *p* = 0.037, Figure 2) effects. Sharing experiences and empathy reduces the caregiver’s perceived burden thereby reducing the intention to engage. At the same time, such sharing has a direct positive effect on the intention to engage.

Both Hypothesis 7 and 8 are supported, showing that caregiver technology affinity (0.458, *p* = 0.004, Figure 2) and ability and openness to seek professional help for psychological diagnoses (1.595, *p* = 0.012, Figure 2) are features of a caregiver that increase intention to engage. The control variable for having a full-time caregiving role is not significant (−0.705, *p* = 0.181, Figure 2).

## 4. Discussion

Our survey takes a cross-sectional view of the caregivers’ existing situation in terms of their personal perception of burden, well-being, and stress, and their caregiving situation in terms of their patient’s and household’s state. The intention to engage measure can be interpreted as a general indication of affinity to receiving help from others, providing help to others, and doing this reciprocally. The presented path model demonstrates how this affinity to exchange support depends on both caregiver and patient characteristics. Within the limitations of our study, the results provide support for the existence of a segment of caregivers that would be willing to engage in a reciprocated exchange.

The variable we propose to measure the intention to engage, ENGAGE, combining existing supply and demand for support along key dimensions with a willingness to reciprocate variable, is reliable and demonstrates face validity. In terms of increasing this intention, the prominent effect of perceived caregiver burden comes as no surprise when burden is viewed as a need for more help. The dependence of burden on caregiver characteristics in the path model is consistent with the literature (34). The strong direct effect of online service or online shopping use suggests that characteristics of caregivers that show technology affinity also make the caregiver more open to exchanging help. Similarly, caregivers seeking help for their psychological problems signal a mindset open to such exchange. Both effects result from caregiver heterogeneity and attempts to influence these can be difficult.

The direct effects of existing support on sharing know-how and sharing caregiver experience and empathy are different in this regard. Sharing know-how, possibly by increasing awareness of the need and possibilities of support, sharing experience and empathy, possibly by increasing mutual understanding, trust, and a sense of community, can increase the intention to engage. Projecting forward, this would suggest that initiatives that improve the existing support landscape of caregivers along the know-how and empathy-sharing dimensions could also instigate openness to other types of exchange. The decrease in intention to engage manifested by the direct effect of help with caregiving tasks (or entrusting the patient) suggests that caregivers who receive support along these dimensions do not perceive a need for further help. Ensuring sustainable exchange along these dimensions will be more challenging on a peer-to-peer platform. However, it may be stimulated via the sharing of know-how, experiences, and empathy. It remains to be explored in future work whether a design that enhances support along certain dimensions enables a further increase in the intention to engage.

Here, caregiver burden was found to be the major factor that affects the intention to engage. A review based on six studies also concluded that caregiver’s technology use and caregiver burden are the major factors that affect adoption of technology (50). Even though current technologies that help for controlling medications, tracking the general health condition of the care recipient and for psychoeducation, can be used to decrease caregiver burden (51), our study adds to that by showing that caregiver burden also increases the intention to engage in a respite platform.

In this study, we did not find an effect of age of the caregiver on the intention to engage. Even though previously, it was reported that non-geriatric caregivers were more willing to use technologies to improve their quality of life (52). In the sample, married caregivers experience lower caregiving burden. This may appear to be in contrast with some of the literature [for example (53)], however married caregivers are not necessarily taking care of their spouses in this sample. We found that chronic social role related stressors and daily activity levels of the care recipient increases caregiver burden and results in increased intention to engage. Similarly, finding resources to understand and cope with the care recipient’s daily dysfunctions was reported to be a major stress relief factor for the caregivers (54).

While it is reassuring to observe that our findings are mostly in line with those in the literature, some limiting aspects of our study should be noted. The use of a convenience sampling approach and the setting in which the survey was conducted may have inherently led to biases in our data. Sampling from caregivers who accompany patients to the clinic may be creating a selection bias. Social desirability and privacy concerns are some of the factors that might have further impacted participation in our study. Travel arrangements and time constraints might have also been influential, perhaps more so on surveys with heavily missing data which have been excluded from the analysis. On the other hand, the burden-centric view of the survey, omitting positive aspects of caregiving may be leading to an underestimation of the intention to engage.

## 5. Conclusion and future perspectives

The presented path analysis reveals that dementia caregivers in the sample demonstrate an overall affinity to the idea of exchanging support with other caregiving households in a reciprocated manner. This provides initial support at the level of individual caregivers to the idea of a peer-to-peer platform where such help can be exchanged. Peer-to-peer exchange enables expanding scarce formal caregiving support services via sharing of informal caregiving resources. Mobilizing informal caregiving resources holds the potential to provide scalable support for family caregivers.

The path model shows that intention to engage in such exchange is affected by the caregiver burden, which in turn further mediates the role of some caregiver and patient-related features on this intention. Caregiver features such as technology affinity and caregiver openness directly affect the intention to engage. The existing support situation of a household is an important driver of the intention to engage.

Our path model displays a good fit, appears to be robust and reveals interesting relationships among several variables. Future work that includes different factors, employs alternative techniques such as a longitudinal approach, and relies on larger and more diverse data sets may help explore the factors that influence intention to engage. It remains to be explored whether a design that enhances support along certain dimensions enables a further increase in the intention to engage.

There are several limitations of our study. The lack of a platform in existence, leads to relying on findings related to similar systems such as time banking systems and hospitality exchange networks to develop our hypotheses. By creating a community, self-reported physical and mental health of time banking participants are found to improve (24). General reciprocity, where members are willing to take from some and give to other members, is indicated as an important factor that determines the success of a time bank (16). The notion of general reciprocity is further combined with offline interaction in online hospitality exchange networks known as couch surfing, where members host some members while staying in the homes of others. Compared to these systems, informal caregivers of dementia patients are a vulnerable group, making safety a critical issue for exchange involving offline interaction. The proposed peer-to-peer platform which has caregiver household-dementia patient dyads acting as peers will require both a commitment to general reciprocity and a much higher level of trust and intimacy (17, 18) to enable offline interactions of caregivers and patients in their homes. This needs to be taken into consideration in future research that addresses the later phases of the system design process. In these phases, the development of a prototype may enable the collection of revealed preferences for a peer-to-peer exchange in a more specific setting, thus alleviating our reliance on self-reported preferences as well. In addition, future studies that focus on the design of the platform may benefit from focus groups and alternative qualitative approaches to support quantitative findings.

With the knowledge that there are caregiver households who would engage in a peer-to-peer exchange, and with a better understanding of causalities, future research may explore whether this segment is large enough and what it implies for the sustainability of such a platform. In our ongoing work, an agent-based simulation model explores the system level feasibility of the proposed concept under different member profiles and platform designs.

Our data collection is performed at a single University Clinic in Turkey via convenience sampling, raising concerns regarding generalizability of our findings. We would like to highlight that the clinic serves as a referral center and thus attracts patients from various backgrounds and a wide geography. Our sample includes caregivers of patients who are at different stages of dementia. Still, our sample may not be representative of the general population in Turkey as these are individuals seeking treatment at a University Clinic. Generalizing our findings to other populations beyond Turkey may be hindered by additional physical and cultural factors such as deficiency in formal care and different social norms. Future multi-center studies that possibly span multiple countries may help alleviate such limitations.

## Data availability statement

The raw data supporting the conclusions of this article will be made available by the authors, without undue reservation.

## Ethics statement

The studies involving human participants were reviewed and approved by Koc University Social Sciences Research Ethics Committee (Decision No: 2017.175.IRB3.088). The patients/participants provided their written informed consent to participate in this study.

## Author contributions

OZA, BB, EG, PG, KK, ELO, SS, and HE: concept and design. OZA, BB, PG, EG, SS, and HE: data collection or processing. OZA, EG, PG, KK, ELO, SS, and HE: analysis or interpretation and literature search. OZA, EG, ELO, SS, and HE: writing. All authors contributed to the article and approved the submitted version.

## Funding

This work was supported by a Koc University internal seed research program fund.

## Conflict of interest

The authors declare that the research was conducted in the absence of any commercial or financial relationships that could be construed as a potential conflict of interest.

## Publisher’s note

All claims expressed in this article are solely those of the authors and do not necessarily represent those of their affiliated organizations, or those of the publisher, the editors and the reviewers. Any product that may be evaluated in this article, or claim that may be made by its manufacturer, is not guaranteed or endorsed by the publisher.
