# Supplementary material for: Caregiver support and burden drive intention to engage in a peer-to-peer exchange of services among caregivers of dementia patients
Source: Front Psychiatry. 2023 Jul 6;14:1208594. doi: 10.3389/fpsyt.2023.1208594 (PMC10359163; doi:10.3389/fpsyt.2023.1208594)
Supplement: Supplementary file 1 [file Data_Sheet_1.pdf]

Supplementary Material

Caregiver support and burden drive intention to engage in a peer-to-peer exchange of services among caregivers of dementia patients

O. Zeynep Aksin *, Basar Bilgic , Perihan Guner , Evrim D. Gunes , Kemal Kuscu , E. Lerzan Ormeci , Serpil Sayin , Hale Yapici Eser

*** Correspondence:**
O. Zeynep Aksin
zaksin@ku.edu.tr

1 Care Environment and Support Assessment Questionnaire

1. The patient;

- Name - Surname: (1) ____
- TR Identity Number: (2) ____

2. How old are you? Please write: ____

3. Your Gender:

- Female (1)
- Male (2)

4. Your education:

- Illiterate (1)
- Literate (2)
- Elementary school (3)
- Middle school (4)
- High school (5)
- University (6)
- Masters degree (7)
- PhD (8)

5. Your marital status:

- single (1)
- married (2)
- Widowed / Divorced (3)

6. Please tick the most appropriate one of the following statements about your employment status.

- I work full time. (1)
- I work part time. (2)
- I am a housewife. (3)
- I am a student. (4)
- I am retired. (5)
- I had to quit my job to take care of my patient. (6)
- Other (please explain) (7) ____

7. How do you evaluate your income?

- Very Low (1)
- Low (2)
- Medium (3)
- High (4)
- Very High (5)

8. Do you have a smartphone?

- Yes (1)
- No (2)

8b. Does anyone have a smartphone at home?

- Yes (1)
- No (2)

9. How is your internet access?

- None (1)
- Restricted (2)
- Sufficient (3)
- Comfortable (4)
- Continuous and Unlimited (5)

9b. (If Your Answer Is No) Does anyone have internet access at home?

- None (1)
- Restricted (2)
- Sufficient (3)
- Comfortable (4)
- Continuous and Unlimited (5)

10. Do you use the applications below?

	Never	Rarely	Sometimes	Often	Always
	(1)	(2)	(3)	(4)	(5)
Whatsapp (1)	○	○	○	○	○
Facebook (2)	○	○	○	○	○
Online Shopping (Hepsiburada, n11.com, etc.) (3)	○	○	○	○	○
Online Services (banking, BiTaksi, e-reservations, etc.) (4)	○	○	○	○	○

11. Your relationship to the patient:

- I'm the spouse (1)
- I'm a child (2)
- I'm a son/daughter in-law (3)
- I’m a sibling (4)
- I'm a relative (5)

- I am a paid assistant (6)
- Other (Please explain): (7) ____

12. How many adults are in your household besides the patient and you?

- Nobody (1)
- 1 (2)
- 2 (3)
- 3 (4)
- 4 or more (5)

13. How are the other adults in the household related to you? (You can mark more than one)

- Spouse (1)
- Child (2)
- Mom or Dad (3)
- Paid helper (4)
- Other (Please explain) : (5) ____

14. For how long have you been caring for your patient?

- less than 1 year (1)
- 1-2 years (2)
- 3-5 years (3)
- 6-10 years (4)
- 11 years and above (5)

15. How many hours a day do you provide care?

- 0-1 hour (1)
- 2-3 hours (2)
- 4-5 hours (3)
- 6-8 hours (4)
- 9-24 hours (5)

16. Do your household support you with household chores?

- Never (1)
- Rarely (2)
- Sometimes (3)
- Often (4)

- Always (5)

17. Can you leave home, entrusting your patient with the parties below?

	Never	Rarely	Sometimes	Often	Always
	(1)	(2)	(3)	(4)	(5)
Family members (1)	○	○	○	○	○
Neighbors / Friends (2)	○	○	○	○	○
Relatives (3)	○	○	○	○	○
Alzheimer Association Volunteer(4)	○	○	○	○	○
Municipal Volunteer (5)	○	○	○	○	○
Private Care Company (6)	○	○	○	○	○
Paid Help (7)	○	○	○	○	○
Other (explain) (8)	○	○	○	○	○

18. Can you get support for caregiving tasks from the parties below while at home?

	Never	Rarely	Sometimes	Often	Always
	(1)	(2)	(3)	(4)	(5)

Family members (1)	○	○	○	○	○
Neighbors / Friends (2)	○	○	○	○	○
Relatives (3)	○	○	○	○	○
Alzheimer Association Volunteer(4)	○	○	○	○	○
Municipal Volunteer (5)	○	○	○	○	○
Private Care Company (6)	○	○	○	○	○
Paid Help (7)	○	○	○	○	○
Other (explain) (8)	○	○	○	○	○

19. Can you receive information or advise on caregiving knowhow from the parties below?

	Never	Rarely	Sometimes	Often	Always
	(1)	(2)	(3)	(4)	(5)

Family members (1)	○	○	○	○	○
Neighbors / Friends (2)	○	○	○	○	○
Relatives (3)	○	○	○	○	○
Alzheimer Association Volunteer(4)	○	○	○	○	○
Municipal Volunteer (5)	○	○	○	○	○
Private Care Company (6)	○	○	○	○	○
Paid Help (7)	○	○	○	○	○
Other (explain) (8)	○	○	○	○	○

20. Can you share experiences and problems related to caregiving with the parties below?

	Never	Rarely	Sometimes	Often	Always
	(1)	(2)	(3)	(4)	(5)

Family members (1)	○	○	○	○	○
Neighbors / Friends (2)	○	○	○	○	○
Relatives (3)	○	○	○	○	○
Alzheimer Association Volunteer(4)	○	○	○	○	○
Paid Help (5)	○	○	○	○	○
Other (explain) (6)	○	○	○	○	○

21. If you had the means, would you like to place your patient in a nursing home?

- I certainly would not (1)
- I would not (2)
- I'm undecided (3)
- I would (4)
- I definitely would (5)

22. What kind of support would you like to receive from a social worker/ volunteer?

	Never	Rarely	Sometimes	Often	Always
	(1)	(2)	(3)	(4)	(5)

Entrust the patient to run errands(1)	○	○	○	○	○
Support in patient care	○	○	○	○	○
Support in household chores (3)	○	○	○	○	○
Information/advise on dementia and patient care (4)	○	○	○	○	○
Information/advise on legal matters related to the patient (5)	○	○	○	○	○
Exchange experiences, chat (6)	○	○	○	○	○

23. What kind of support would you like to receive from someone with a dementia patient in their family?

	Never	Rarely	Sometimes	Often	Always
	(1)	(2)	(3)	(4)	(5)

Entrust the patient to run errands(1)	○	○	○	○	○
Support in patient care	○	○	○	○	○
Support in household chores (3)	○	○	○	○	○
Information/advise on dementia and patient care (4)	○	○	○	○	○
Exchange experiences, chat (5)	○	○	○	○	○

24. What kind of support would you like to receive from a healthcare professional?

	Never	Rarely	Sometimes	Often	Always
	(1)	(2)	(3)	(4)	(5)
Entrust the patient to run errands(1)	○	○	○	○	○
Support in patient care	○	○	○	○	○
Information/advise on dementia and patient care (3)	○	○	○	○	○

25. What kind of support can you provide to someone with a dementia patient in their family?

	Never	Rarely	Sometimes	Often	Always
	(1)	(2)	(3)	(4)	(5)
Entrust the patient to run errands(1)	○	○	○	○	○
Support in patient care	○	○	○	○	○
Support in household chores (3)	○	○	○	○	○
Information/advise on dementia and patient care (4)	○	○	○	○	○
Exchange experiences, chat (5)	○	○	○	○	○

26. What kind of support can someone in your household provide to someone with a dementia patient in their family?

	Never	Rarely	Sometimes	Often	Always
	(1)	(2)	(3)	(4)	(5)
Entrust the patient to run errands(1)	○	○	○	○	○
Support in patient care	○	○	○	○	○
Support in household chores (3)	○	○	○	○	○
Information/advise on dementia and patient care (4)	○	○	○	○	○
Exchange experiences, chat (5)	○	○	○	○	○

27. What would your expectations be in return for such services?

	Definitely do not agree	Do not agree	undecided	Agree	Definitely agree
	(1)	(2)	(3)	(4)	(5)

Nothing (1)	o	o	o	o	o
To receive similar services (2)	o	o	o	o	o
Monetary compensation (3)	o	o	o	o	o

28. Do you have any physical or mental illness diagnosed by a doctor?

- Yes (1)
- No (2)

28b. If your answer is Yes, please specify.

____

29. If you have a diagnosed physical illness, when was this diagnosis made?

- After starting to care for the patient (1)
- Before starting to care for the patient (2)

30. If you have a diagnosed mental/psychological disease, when was this diagnosis made?

- After starting to care for the patient (1)
- Before starting to care for the patient (2)

31. Do you have any medication that you constantly use for your mental / psychological problems?

- Yes (1)
- No (2)

32. If you are using medication for a mental/psychological problem, it started ___

- After I started caring for my patient (1)
- Before starting to care for the patient (2)

33. Do you receive any psychological help?

- Yes (1)
- No (2)
